# Chronic and Acute Ozone Exposure in the Week Prior to Delivery Is Associated with the Risk of Stillbirth

**DOI:** 10.3390/ijerph14070731

**Published:** 2017-07-06

**Authors:** Pauline Mendola, Sandie Ha, Anna Z. Pollack, Yeyi Zhu, Indulaxmi Seeni, Sung Soo Kim, Seth Sherman, Danping Liu

**Affiliations:** 1 Division of Intramural Population Health Research, Eunice Kennedy Shriver National Institute of Child Health and Human Development, 6710B Rockledge Drive, Bethesda, MD 20892, USA; sha55@ucmerced.edu (S.H.); indu.seeni@nih.gov (I.S.); danping.liu@nih.gov (D.L.); 2 Department of Global and Community Health, College of Health and Human Services, George Mason University, 4400 University Drive/MS5B7, Fairfax, VA 22030, USA; apollac2@gmu.edu; 3 Division of Research, Kaiser Permanente Northern California, 2000 Broadway, Oakland, CA 94612, USA; yeyi.zhu@kp.org; 4 Center for Genome Science, National Research Institute of Health, OHTAC, 200 Osongsaengmyeong2-ro, Osong-eup, Heungdeok-gu, Cheongju-si, Chungcheongbuk-do 28160, Korea; ksungsoo@korea.kr; 5 The Emmes Corporation, 401 N Washington St # 700, Rockville, MD 20850, USA; ssherman@emmes.com

**Keywords:** stillbirth, ozone, air pollution, pregnancy

## Abstract

Chronic and acute air pollution has been studied in relation to stillbirth with inconsistent findings. We examined stillbirth risk in a retrospective cohort of 223,375 singleton deliveries from 12 clinical sites across the United States. Average criteria air pollutant exposure was calculated using modified Community Multiscale Air Quality models for the day of delivery and each of the seven days prior, whole pregnancy, and first trimester. Poisson regression models using generalized estimating equations estimated the relative risk (RR) of stillbirth and 95% confidence intervals (CI) in relation to an interquartile range increase in pollutant with adjustment for temperature, clinical, and demographic factors. Ozone (O_3_) was associated with a 13–22% increased risk of stillbirth on days 2, 3, and 5–7 prior to delivery in single pollutant models, and these findings persisted in multi-pollutant models for days 5 (RR = 1.22, CI = 1.07–1.38) and 6 (RR = 1.18, CI = 1.04–1.33). Whole pregnancy and first trimester O_3_ increased risk 18–39% in single pollutant models. Maternal asthma increased stillbirth risk associated with chronic PM_2.5_ and carbon monoxide exposures. Both chronic and acute O_3_ exposure consistently increased stillbirth risk, while the role of other pollutants varied. Approximately 8000 stillbirths per year in the US may be attributable to O_3_ exposure.

## 1. Introduction

Ambient air pollution exposure is associated with a variety of adverse pregnancy outcomes including infant mortality, preterm birth, and impaired fetal growth [1]. Spontaneous abortion (pregnancy loss before 20 weeks) also appears to be increased after air pollutant exposure [2], and these early losses may share a common pathway with stillbirth. A recent systematic review of the role of prenatal ambient air pollution concluded that exposure appeared to increase stillbirth risk, particularly after late pregnancy exposure to sulfur dioxide (SO_2_), carbon monoxide (CO), particulate matter ≤10 microns (PM_10_), and ozone (O_3_), although most meta-analysis results included the null [3]. The meta-analyses were limited by crude exposure assessments based on ambient monitors and a reliance on registry data with limited information on important confounders and effect modifiers [4]. One important but understudied potential modifier is maternal asthma. A recent meta-analysis found that maternal asthma was associated with a 25% increased risk of perinatal mortality, which reflects a combination of neonatal death and stillbirth, although stillbirth alone was not significantly elevated among asthmatics [5]. 

Only two studies have examined the acute stillbirth risk associated with exposures in the week prior to delivery, with somewhat conflicting results. A time-series analysis from Sao Paulo, Brazil in the late 1990s found nitrogen dioxide (NO_2_) exposure had the strongest relation to increased stillbirth risk, but stillbirth was also associated with an overall pollution summary score, SO_2_, and CO, while no increased risk was observed for PM_10_ or O_3_ [6]. Vital records data from New Jersey (1998–2004) were used in a case-crossover study that also found an acute increased stillbirth risk associated with SO_2_ and CO (in this case two to three days prior to delivery), but no relation with NO_2_ or PM ≤ 2.5 microns (PM_2.5_) [7]. 

Stillbirth is an important and preventable global concern [8] with a prevalence of up to 3% in some regions of the world [9], yet this research area receives low priority on the global health agenda [10]. Some known contributors to stillbirth risk include genetics, childbirth complications, pregnancy complications (e.g., preeclampsia), fetal growth restriction, and congenital abnormalities [11,12]. Many of these factors are not easily amenable to intervention, whereas ambient air pollution exposure is potentially modifiable. Because these exposures are ubiquitous, even small increases in risk can have large effects on a population level. 

The purpose of this study was two-fold. First, we aimed to determine the associations between chronic and acute exposures to common ambient air pollutants and stillbirth risk in a large contemporary US obstetric cohort with modeled exposure assessment and medical record data on potential confounders and effect modifiers, including maternal asthma status. Second, we estimated the excess number of stillbirths potentially attributable to air pollution exposure in the US.

## 2. Materials and Methods

### 2.1. Study Population

The Consortium on Safe Labor (CSL) is a retrospective cohort of 228,438 deliveries between 2002–2008 from 12 clinical centers across the US [13]. Data on maternal demographic characteristics; medical, reproductive, and prenatal history; labor and delivery; and postpartum and newborn information were extracted from electronic medical records for births at 23 weeks of gestation or later. Electronic discharge summaries for mothers and infants were linked to the medical records. Data for these analyses were limited to 223,375 singleton pregnancies among 204,165 women. Institutional review board approval was obtained at all participating institutions. Since this study represented a retrospective review of electronic medical records, it was classified as exempt by the Office of Human Subjects Research (OHSR) at the National Institutes of Health (Exempt #3854, 2007). Data are anonymized and publicly available [14]. 

### 2.2. Outcome

Stillbirth was defined as any fetal death ≥23 weeks of gestation reported in electronic medical records supplemented with ICD-9 codes in hospital discharge summaries. Both intrapartum and antepartum deaths are recorded. Due to the lack of data on the timing of antepartum stillbirth, the date of delivery was used as a proxy for event time. 

### 2.3. Exposure 

Estimates of ambient air pollutant exposures for all pregnancies in the CSL were quantified as part of the Air Quality and Reproductive Health study. Exposures of interest for these analyses are criteria air pollutants including CO, O_3_, nitric oxides (NO_x_), PM_10_, PM_2.5_, and SO_2_. Preliminary exploration of PM_2.5_ constituents and air toxics were generally null and not pursued. Modified Community Multiscale Air Quality (CMAQ) models calculated hourly air pollution exposure based on reported air pollution emissions and accounting for the effects of weather, and chemical reactions between pollutants on the exposure [15]. Meteorology inputs and pollutant emissions used in the model simulations were obtained from the Weather Research and Forecasting model and the National Emission Inventories, respectively [16]. Since the outcome data are anonymized, exposures were estimated based on each hospital referral region. Hourly air pollutant concentration predictions were weighted by population density within the hospital referral region to account for places where women were unlikely to live and work [15]. Modeled data were also fused with observed monitor data from the US Environmental Protection Agency’s Air Quality System to correct for measurement error between modeled and observed values [15]. Finally, hourly estimates of air pollution were averaged to obtain individual daily means for women across their pregnancy and averaged up for longer time windows. 

Chronic exposures were assessed as daily average over the whole pregnancy (week 1 through birth) and the first trimester (weeks 1–13). Only the first trimester was assessed because a significant proportion of stillbirths (33%, or 328 of 992) were delivered before the completion of the second trimester. Acute exposures of interest included daily average concentration of pollutants during the day of delivery and each of the seven days before delivery.

### 2.4. Covariates

Maternal age in years (<20, 20–24, 25–29, 30–34, ≥35, unknown); race/ethnicity (Non-Hispanic White, Non-Hispanic Black, Hispanic, Other, Unknown), pre-pregnancy body mass index (BMI, kg/m^2^; <18.5 underweight, 18.5–24.9 normal weight, 25–29.9 overweight, ≥30 obese, unknown), smoking and alcohol use during pregnancy (both yes, no/unknown), parity (nulliparous, primiparous, multiparous), insurance status (private, public/government, other, unknown), marital status (married, not married, unknown), and maternal pre-existing hypertension, asthma, and diabetes were derived from the medical record. Analyses were also adjusted for study site, birth year, average ambient temperature during each time window of interest, and season of conception. Selection of covariates was based on our previous studies of maternal asthma and obstetric outcomes [17] and stillbirth in relation to temperature [18] in this cohort.

### 2.5. Statistical Analysis

Pregnancy is the unit of analysis for all statistical testing. Frequency distributions for covariates were calculated for pregnancies with and without stillbirth. Poisson regression models estimated the relative risk (and 95% confidence interval) of stillbirth associated with average pollutant exposure during the whole pregnancy, the first trimester, the day of delivery, and each of the seven days preceding delivery. Since some women (8.6%) contributed more than one pregnancy to the cohort, the within-subject correlation was accounted for by using the robust variance estimation in generalized estimating equations. Pregnancies that resulted in live birth were the comparison group. Pollutant exposures were analyzed in continuous scale, and the relative risks were calculated per interquartile range (IQR; the difference between the 25th and 75th percentile) increase in each pollutant. We also evaluated quadratic terms to test our linearity assumption. No quadratic terms were significant, so linear models were used.

For each time window of interest, we fit a single-pollutant model adjusted for covariates including maternal age, maternal race, marital status, parity, pre-pregnancy body mass index, insurance, maternal smoking during pregnancy, maternal alcohol use during pregnancy, pre-existing hypertension or diabetes, birth year, ambient temperature, and season of conception. Multi-pollutant models included all criteria pollutants in addition to the above covariates. 

We conducted analyses to assess the robustness of our whole pregnancy exposure measure considering pregnancies of varying lengths and to evaluate effect modification. For the whole pregnancy exposure analysis, to ensure comparable length of exposure, we stratified stillbirths by gestational age and truncated the exposures among ongoing births as a comparison. Specifically, due to the small number of stillbirths at each gestational age, we divided births into several strata (weeks 23–26, 27–30, 31–36, and 37–42). For stillbirths that were delivered during weeks 23–26, we compared their whole pregnancy exposures to ongoing pregnancies with exposures truncated at 23 weeks. This same method was applied to births in other strata. A secondary analysis also examined antepartum and intrapartum stillbirths separately. Interactions between maternal asthma and air pollution in relation to stillbirth risk were also explored, as some of our previous work has shown a differential pattern of risk associated with ambient air pollutants for asthmatics [19,20].

We calculated the attributable risk (AR) to estimate the number of excess stillbirth cases per 100,000 births associated with an IQR increase in O_3_ exposure using the following formula:

(1) $$AR=I_{e}-I_{u}$$

where $I_{e}$ stands for the incidence among the exposed and $I_{u}$ stands for background incidence. $I_{e}$ was calculated as the background incidence $I_{u}$ times the RR for an IQR increase in pollutants. We used two measures for $I_{u}$: our study-specific background incidence (0.44%), and the U.S. background incidence of stillbirths from 2002 to 2008 (0.62%) based on the National Vital Statistics Report [21]. All analyses were conducted using SAS 9.4 (SAS, Cary, NC, USA). 

## 3. Results

Among 223,375 singleton births in our cohort, there were 992 cases of stillbirth (0.44%). Table 1 shows that stillbirth was more common among mothers who were <20 or ≥35 years old, non-Hispanic Black, not married, nulliparous, smoked or consumed alcohol during pregnancy, or had preexisting hypertension or diabetes. Women with asthma had a slightly higher rate of stillbirth, 0.52% compared to 0.44% for non-asthmatics (*p* = 0.14). Concentrations of air pollutants appeared to be similar between stillbirths and livebirths across all exposure windows (Supplemental Table S1). The distribution of air pollutants across all windows and their Spearman coefficients of correlation are presented in Supplemental Tables S2 and S3, respectively. Overall, the levels of exposure in all time windows were fairly moderate, and when averaged over time did not exceed US ambient air quality standards. 

Both chronic and acute prenatal exposures to O_3_ were associated with increased risk of stillbirth (Table 2). Specifically, an interquartile range increase in average daily O_3_ exposure during the first trimester and whole pregnancy was associated with 18% (95% CI: 0–39%) and 39% (5–84%) increased stillbirth risk, respectively. Using the U.S. background incidence of stillbirth as reference [21], these risks are equivalent to an excess of about 111 and 241 excess cases per 100,000 births for the first trimester and whole pregnancy exposures, respectively (Table 3).

Acute exposures during the week prior to delivery were also associated with risk, particularly for exposures 2, 3, and 5–7 days prior (Table 2). For example, an IQR increase in O_3_ two days before delivery was associated with 15% (95% CI: 1–31%) increased risk, which is equivalent to about 95 excess cases of stillbirth per 100,000 births based on the national annual incidence. This translates to an 8% (95% CI: 1–16%) increased risk based on a 10 ppb increase in ozone two days before delivery. During the other days prior to delivery, the increased risk associated with a 10 ppb increase in ozone ranges from 7–12%. Other pollutants did not show significant associations, except for CO and SO_2_ exposure during the first trimester, both of which showed inverse associations with about 20% decreased risk for an IQR increase. Analyses with truncated whole-pregnancy exposures for O_3_ had generally similar results, but confidence intervals were widened due to low sample size in each time stratum (Supplemental Table S4). Multi-pollutant models were also generally consistent, but confidence intervals were widened during some windows, potentially due to collinearity among pollutants as well as the loss of precision due to additional covariates. In the multipollutant models, an IQR increase PM_2.5_ exposures during the day before delivery was associated with 15% (95% CI: 1–31%) increased stillbirth risk, which was not observed in the single-pollutant model (Supplemental Table S5). 

Point estimates for antepartum (*n* = 896) and intrapartum (*n* = 96) stillbirth analyses were very similar, although the confidence limits for intrapartum results included the null due to the smaller sample size (data not shown). Asthma significantly modified the stillbirth risk (Table 4) associated with an IQR increase in whole pregnancy PM_2.5_ (RR = 1.21 vs. 1.15, and first trimester average exposure to PM_2.5_ (RR = 1.08 vs. 0.99), SO_2_ (RR = 0.99 vs. 0.89), NO_x_ (RR = 0.99 vs. 0.92), and CO (RR = 1.11 vs. 0.94). Effect modification by asthma was not significant for O_3_ and PM_10_, although O_3_ had a main effect on stillbirth for both asthmatics and non-asthmatics. Acute exposure risk in the week prior to delivery did not differ by asthma status (Supplemental Table S6).

## 4. Discussion

We found that both chronic and acute O_3_ exposures in the days prior to delivery were associated with increased stillbirth risk in a large US obstetric cohort. We previously found increased stillbirth risk related to chronic exposure to temperature extremes and acute changes in absolute temperature [18], and these O_3_ findings remain consistent after adjustment for ambient temperature and other important clinical covariates. This study represents a step forward in the literature, which has previously relied heavily on vital records and relatively crude exposure assessment with ambient air monitors. In our final models, first trimester CO and SO_2_ exposures were inversely associated with risk, but no consistent effects were seen for NO_x_, PM_10_, or PM_2.5_. Although the numbers were small, we did observe that women with asthma had a slightly higher stillbirth risk and some pollutants—particularly first trimester and whole pregnancy PM_2.5_ and whole pregnancy CO—appeared to differentially increase the risk among asthmatics compared to non-asthmatics. Our findings suggest that approximately 200 stillbirths per 100,000 births each year might be attributable to chronic ozone exposure in the US. This translates to approximately 8000 potentially preventable stillbirths annually (given four million births per year) if our findings were confirmed. 

The literature on chronic exposure to ambient air pollutants and stillbirth is quite mixed, and some early studies were completely null. No effects on stillbirth in the Czech Republic were observed in the late 1980s in relation to the annual geometric mean of SO_2_, NO_x_, and suspended particles [22]. A Swedish study (1985–1990) also found no effect associated with chronic SO_2_ or NO_x_ exposures [23], and no effect was observed related to black smoke exposure in the northern area of England (1962–1992) [24]. Notably, these studies relied on crude exposure metrics, and it is possible that lack of specificity in exposure measurement contributed to the lack of association that was observed. 

More recent studies find sporadic effects for particulate matter. For example, PM_2.5_ has been studied using data from Ohio (2006–2010), and while continuous exposure measures were not related to stillbirth risk, high exposure in the third trimester (generally >16 µg/m^3^) increased risk [25]. A hospital-based study of PM_10_ and adverse pregnancy outcomes observed that third trimester exposure increased stillbirth risk in Seoul [26]. In contrast, a study in New Jersey (1998–2004) found chronic exposure to NO_2_, SO_2_, and CO appeared to increase stillbirth risk, but no increased risks were observed with PM_2.5_ [27]. We observed an increased risk associated with PM_2.5_ exposure for the day prior to delivery in a multi-pollutant model that was not apparent in the single-pollutant model, suggesting that the result might be due to the correlation of PM_2.5_ with other pollutants. 

With regard to other gaseous criteria air pollutants, our findings for O_3_ are consistent with a population-based study in California (1999–2009) that observed an association between late pregnancy O_3_ and stillbirth, but were not consistent with respect to whole pregnancy NO_2_, which was significant in that report [28]. SO_2_ and PM_10_ during early pregnancy appeared to increase risk in the Taiwanese population (2001–2007) with no effect observed for O_3_, CO, or NO_2_ [29], which is also in contrast to our generally null findings with the exception of O_3_. Our findings with respect to O_3_ and stillbirth were consistent with a recent meta-analysis in China, although our findings indicated a stronger association [30]. The inverse associations we observed with first trimester CO and SO_2_ are contrary to our hypotheses. Both are negatively correlated with ozone, which may account for some of the association, although the findings remain in multipollutant models. It appears that endogenous SO_2_ can inhibit oxidative stress in a rodent model [31], but it is unclear whether ambient levels of SO_2_ among humans would operate in a similar fashion. 

We join a small number of studies that have examined the potential acute triggering of stillbirth after exposures in the days prior to delivery. In contrast to the study by Faiz and colleagues [7], we did not observe an acute effect for SO_2_ or CO, but our null findings for PM_2.5_ and NO_2_ are similar; they did not report on O_3_. An older time series study [6] did report an acute effect for NO_2_, SO_2_, and CO, but not for PM_10_ or O_3_. 

Our study has several limitations as well as strengths associated with both our clinical and air pollution model data. First, we recognize that intrapartum stillbirths are more likely related to complications of delivery [32] and perhaps less likely to be influenced by ambient exposures. As such, our similar effect estimates for intrapartum and antepartum deaths suggest that some risks may be related to complications with a shared longer-term pathway. Unfortunately, we lack statistical power to examine intrapartum deaths in more detail, and the cause of death is unavailable in our anonymized data. We also acknowledge that antepartum timing of death is unavailable and generally thought to precede delivery by about 48 h. This makes our acute findings for days 2–3 and 5–6 more plausible as potential triggering events. Air pollution is known to generate reactive oxygen species, which are implicated in placental insufficiency that can lead to fetal demise [33]. Further, increased plasma viscosity has been found with exposure to air pollution, reflecting an additional biological pathway that could play a role in stillbirth [34]. Our data are also limited by the medical records available, hospital deliveries at 23 weeks gestation or later to support the original study objective of assessing contemporary labor and delivery obstetric practice [13]. While this rich clinical data allows us to control for a variety of important clinical factors which are typically unavailable in studies based on vital records, we have no information on losses prior to 23 weeks. This difference in coverage is likely a partial explanation for our lower estimate of stillbirth (444.1 cases per 100,000 births) compared to the US rates which are based on stillbirths at 20 weeks or later (622.3 cases per 100,000 births). We are also unable to investigate spontaneous abortion (which occurs prior to 20 weeks) in these data. A recent review suggests that spontaneous abortion may be increased by air pollutant exposure, but the data are relatively sparse [2]. One ecological study found that monthly levels of ozone and PM_10_ were associated with city-wide increased spontaneous abortion rates, but no association was observed for NO_2_ [35]. Given potential risks across gestation, the overall risk of pregnancy loss associated with ozone exposure may be higher than we observed, and further large-scale clinical studies that can explore this full spectrum of effects are needed. With regard to exposure assessment, we make use of a state-of-the-art modeling process to estimate exposures, but due to the anonymous nature of our clinical data, the exposures are calculated as averages over the delivery hospital referral region. While this will add to non-differential misclassification, we expect that this would drive estimates towards the null and that our estimates are conservative in that sense. The broader exposure may help us account for the time women are away from their residences in activities of daily living. We take the additional step of estimating the population attributable risk (AR) because although the risk estimates for ozone are modest, the exposures potentially impact a large population. However, we are cautious in our interpretation of the AR, since the underlying assumptions are a causal relationship with no residual confounding. Finally, we were able to make use of a very rich clinical data source to adjust for many confounders and potential effect modifiers, something that is generally lacking in prior studies based on vital records. Even with this detailed data, we lacked information on asthma severity or treatment, which may have influenced the effect modification findings. As such, our findings represent an average risk associated with maternal asthma, and further research on women with poorly controlled asthma may be warranted. Our findings suggest that women with asthma have higher rates of stillbirth, and they may be more susceptible to air pollution exposure compared to non-asthmatics.

## 5. Conclusions

In this nationwide US obstetric cohort based on electronic medical record data with air pollution estimated by modified Community Multiscale Air Quality models, stillbirth risk was increased after both chronic and acute O_3_ exposure. Ozone exposure in the week prior to delivery was associated with a 13–22% increased stillbirth risk, while chronic exposure over the course of pregnancy increased risk by nearly 40%. PM_2.5_ exposure in the day prior to delivery also appeared to increase stillbirth risk in multi-pollutant models. Although the number of cases was small, maternal asthma modified stillbirth risk associated with chronic PM_2.5_ and CO exposures. If our findings were confirmed, they suggest that approximately 8000 stillbirths per year in the US could be attributable to O_3_ exposure during pregnancy.

## Figures and Tables

**Table 1:** Characteristics of pregnancies by stillbirth status from the Consortium on Safe Labor, 2002–2008 (*n* = 223,375).

Characteristics	No Stillbirth *n* = 222,383		Stillbirth *n* = 992		*p*-Value ^a^
	*n*	%	*n*	%	
Maternal age					0.014
<20	20,574	9.3	121	12.2	
20–24	56,333	25.3	248	25	
25–29	61,982	27.9	230	23.2	
30–34	49,947	22.5	198	20	
≥35	33,242	15	193	19.5	
Unknown	305	0.1	2	0.2	
Maternal race					<0.001
NH-White	110,216	49.6	325	32.8	
NH-Black	49,917	22.5	338	34.1	
Hispanic	38,628	17.4	183	18.5	
Other	14,347	6.5	58	5.9	
Unknown	9275	4.2	88	8.9	
Marital status					<0.001
Married	130,728	58.8	447	45.1	
Not married	84,530	38	464	46.8	
Unknown	7125	3.2	81	8.2	
Parity					0.001
0	88,580	39.8	444	44.8	
1	68,151	30.7	238	24	
≥2	65,652	29.5	310	31.3	
Pre-pregnancy BMI					<0.001
<18.5	7950	3.6	26	2.6	
18.5–24.9	78,773	35.4	268	27	
25–29.9	33,351	15	144	14.5	
≥30	27,732	12.5	143	14.4	
Unknown	74,577	33.5	411	41.4	
Insurance type					<0.001
Private	124,459	56	444	44.8	
Public	71,829	32.3	318	32.1	
Other	2963	1.3	19	1.9	
Unknown	23,132	10.4	211	21.3	
Smoking	14,834	6.7	96	9.7	0.002
Alcohol	4058	1.8	32	3.2	0.013
Pre-existing Hypertension	4311	1.9	51	5.1	<0.001
Pre-Existing Diabetes	3261	1.5	48	5.1	<0.001
Asthma	16,860	7.8	88	8.9	0.139
Season of conception					0.056
Spring (March–May)	52,615	23.7	253	25.5	
Summer (June–August)	57,450	25.8	214	21.6	
Fall (September–November)	61,002	27.4	257	25.9	
Winter (December–February)	51,316	23.1	268	27	

Abbreviation: BMI, body mass index; NH, non-Hispanic; ^a^ Obtained from generalized estimating equations to account for women who had more than one pregnancy in the study period.

**Table 2:** Risk of stillbirth related to acute and chronic criteria air pollutant exposures.

Adjusted Relative Risk (95% CI) ^a^						
Exposure Windows	Ozone	CO	NO_x_	PM_10_	PM_2.5_	SO_2_
Days before delivery						
0 (delivery day)	1.09 (0.96,1.24)	0.97 (0.88, 1.07)	1.01 (0.93, 1.11)	0.98 (0.89, 1.08)	0.97 (0.88, 1.06)	0.99 (0.91, 1.07)
1	1.08 (0.95, 1.23)	1.01 (0.92, 1.11)	1.06 (0.98, 1.16)	1.05 (0.96, 1.15)	1.00 (0.92, 1.1)	1.00 (0.93, 1.08)
2	**1.15 (1.01**, **1.31)**	0.94 (0.85, 1.04)	0.96 (0.88, 1.04)	1.06 (0.98, 1.16)	1.03 (0.95, 1.12)	1.02 (0.95, 1.09)
3	**1.13 (1.00**, **1.29)**	0.92 (0.83, 1.02)	0.95 (0.86, 1.03)	1.08 (0.99, 1.18)	1.06 (0.98, 1.15)	1.02 (0.95, 1.09)
4	1.12 (0.99, 1.27)	1.01 (0.91, 1.11)	1.00 (0.92, 1.10)	1.02 (0.94, 1.11)	1.03 (0.95, 1.11)	0.99 (0.93, 1.06)
5	**1.22 (1.07**, **1.38)**	0.97 (0.88, 1.07)	0.96 (0.88, 1.06)	0.96 (0.87, 1.05)	0.99 (0.91, 1.08)	0.99 (0.92, 1.07)
6	**1.18 (1.04**, **1.33)**	1.02 (0.92, 1.12)	1.01 (0.92, 1.10)	1.04 (0.95, 1.13)	1.07 (0.99, 1.16)	1.04 (0.97, 1.12)
7	**1.14 (1.01**, **1.30)**	1.00 (0.91, 1.10)	1.01 (0.92, 1.10)	1.04 (0.96, 1.14)	1.05 (0.96, 1.14)	0.97 (0.89, 1.05)
First trimester	**1.18 (1.00**, **1.39)**	**0.80 (0.68**, **0.94)**	0.85 (0.66, 1.09)	0.97 (0.85, 1.12)	0.91 (0.77, 1.07)	**0.80 (0.66**, **0.96)**
Whole pregnancy	**1.39 (1.05**, **1.84)**	0.87 (0.70, 1.08)	0.96 (0.62, 1.49)	1.04 (0.86, 1.25)	1.08 (0.75, 1.55)	0.79 (0.54, 1.15)

Abbreviations: CI, confidence interval; CO, carbon monoxide; NO_x_, nitric oxides; PM_10_, particulate matter with diameter <10 microns; PM_2.5,_ particulate matter with diameter <2.5 microns; SO_2_, sulfur dioxide; Bold face indicates statistical significance at *p* < 0.05; ^a^ Models adjusted for maternal age, maternal race, parity, smoking and alcohol use during pregnancy, insurance status, marital status, pre-existing hypertension, pre-existing diabetes, season of conception, birth year, site, and average temperature.

**Table 3:** Annual excess number of stillbirths (per 100,000 births per year) associated with exposures to ozone.

Exposure Windows	AR (95% CI) ^a^	AR (95% CI) ^b^
Days before delivery		
0 (delivery day)	42.1 (−16.0, 108.1)	59.0 (−22.5, 151.5)
1	35.0 (−22.4, 100.1)	49.0 (−31.4, 140.2)
2	**67.9 (6.4**, **137.8)**	**95.2 (9.0**, **193.2)**
3	59.1 (−2.0, 128.7)	82.8 (−2.8, 180.4)
4	54.8 (−4.4, 121.9)	76.7 (−6.1, 170.8)
5	**97.3 (32.6**, **170.8)**	**136.3 (45.7**, **239.3)**
6	**78.6 (16.8**, **148.7)**	**110.1 (23.6**, **208.3)**
7	**64.3 (3.0**, **133.9)**	**90.1 (4.2**, **187.7)**
First trimester	**79.1 (0.3**, **171.9)**	**110.8 (0.4**, **240.8)**
Whole pregnancy	**172.1 (21.9**, **370.9)**	**241.2 (30.7**, **519.7)**

Abbreviations: AR, attributable risk; CI, confidence interval; Bold face indicates statistical significance at *p* < 0.05. ^a^ Calculated based on study specific background incidence of 444.1 cases per 100,000 births (2002–2008); ^b^ Calculated based on estimated US background incidence of 622.3 cases per 100,000 births during the study period 2002–2008 (estimated by the National Vital Statistic Reports [21].

**Table 4:** Adjusted ^a^ relative risk (and 95% confidence intervals) for stillbirth per interquartile range (IQR) unit increase in pollutant by exposure window and asthma status.

Pollutant	Asthma Status	Trimester 1	Whole Pregnancy
		RR (99% CI)	RR (99% CI)
CO	Asthma	**1.11 (0.80**, **1.54)**	1.04 (0.72, 1.50)
	No asthma	**0.94 (0.78**, **1.13)**	0.95 (0.76, 1.18)
NO_X_	Asthma	**0.99 (0.67**, **1.46)**	1.09 (0.64, 1.88)
	No asthma	**0.92 (0.71**, **1.19)**	1.07 (0.68, 1.68)
O_3_	Asthma	1.00 (0.73, 1.38)	1.22 (0.81, 1.85)
	No asthma	1.09 (0.90, 1.32)	1.25 (0.91, 1.70)
SO_2_	Asthma	**0.99 (0.70**, **1.40)**	1.09 (0.67, 1.77)
	No asthma	**0.89 (0.71**, **1.11)**	0.97 (0.66, 1.43)
PM_10_	Asthma	0.97 (0.72, 1.31)	0.92 (0.69, 1.22)
	No asthma	0.97 (0.81, 1.16)	0.95 (0.78, 1.15)
PM_2.5_	Asthma	**1.08 (0.77**, **1.52)**	**1.21 (0.76**, **1.92)**
	No asthma	**0.99 (0.80**, **1.22)**	**1.15 (0.80**, **1.65)**

Boldface indicates significant interaction between maternal asthma and pollutant at *p* < 0.05; Abbreviations: RR, relative risk; CI, confidence interval; CO, carbon monoxide; NO_x_, nitric oxides; PM_10_, particulate matter with diameter <10 microns; PM_2.5_, particulate matter with diameter <2.5 microns; SO_2_, sulfur dioxide; ^a^ Models adjusted for maternal age, maternal race, parity, smoking and alcohol use during pregnancy, insurance status, marital status, pre-existing hypertension, pre-existing diabetes, season of conception, birth year, site, and average temperature. Estimates are for an IQR increase in each pollutant.

## Supplementary Materials

The following are available online at www.mdpi.com/1660-4601/14/7/731/s1. Table S1: Distribution of air pollutants by stillbirth status, Table S2: Distribution of air pollutants by exposure windows, Table S3: Pearson correlation coefficient among pollutants during the whole pregnancy period; Table S4: Associations between whole pregnancy exposures to criteria air pollutants and stillbirth risk by gestational week; Table S5: Multipollutant associations between whole pregnancy exposures to criteria air pollutants and stillbirth risk; Table S6: Adjusted relative risk (and 95% confidence intervals) for stillbirth per IQR unit increase in pollutant by exposure window and asthma status.
