# Supplementary material for: Chronic and Acute Ozone Exposure in the Week Prior to Delivery Is Associated with the Risk of Stillbirth
Source: Int J Environ Res Public Health. 2017 Jul 6;14(7):731. doi: 10.3390/ijerph14070731 (PMC5551169; doi:10.3390/ijerph14070731)
Supplement: Supplementary file 1 [file ijerph-14-00731-s001.pdf]

Supplemental Table 1. Distribution of air pollutants by stillbirth status.

Exposure window	Pollutant[a]	No Stillbirth Mean (SD)	Stillbirth Mean(SD)
Delivery day 0	CO	529.7 (246.9)	541.7 (236.5)
	NO_x	29.7 (27.4)	29.5 (28.9)
	O_3	29.9 (11.8)	29.2 (11.7)
	PM_{10}	22.5 (11.1)	22.1 (11.1)
	$PM_{2.5}$	11.8 (7.4)	11.7 (7.7)
	SO_2	3.9 (3.5)	3.8 (3.4)
Days prior to delivery			
1	CO	528 (245.3)	545.3 (235.2)
	NO_x	29.4 (27.2)	30.0 (29.5)
	O_3	30.0 (11.8)	29.3 (11.6)
	PM_{10}	22.4 (11.1)	22.6 (11.6)
	$PM_{2.5}$	11.8 (7.4)	11.9 (7.9)
	SO_2	3.9 (3.5)	3.8 (3.2)
2	CO	526.0 (245.4)	534.5 (227.2)
	NO_x	29.0 (27.0)	28.1 (25.7)
	O_3	30.1 (11.8)	29.7 (11.7)
	PM_{10}	22.3 (11.1)	22.7 (11.1)
	$PM_{2.5}$	11.8 (7.4)	12.1 (7.7)
	SO_2	3.9 (3.5)	3.8 (3.3)
3	CO	525.0 (243.8)	530.7 (221.9)
	NO_x	28.7 (26.5)	27.5 (25.4)
	O_3	30.1 (11.8)	29.7 (12.0)
	PM_{10}	22.2 (11.0)	22.6 (11.1)
	$PM_{2.5}$	11.8 (7.3)	12.2 (7.6)
	SO_2	3.9 (3.5)	3.8 (3.3)
4	CO	525.3 (243.5)	542.1 (235)
	NO_x	28.7 (26.4)	28.4 (27.6)
	O_3	30.1 (11.8)	29.6 (11.7)
	PM_{10}	22.2 (11.0)	22.2 (10.5)
	$PM_{2.5}$	11.8 (7.3)	12.0 (7.3)
	SO_2	3.9 (3.5)	3.8 (3.1)
5	CO	526.4 (243.6)	538 (232.9)
	NO_x	28.9 (26.6)	27.9 (26.7)
	O_3	30.1 (11.8)	29.9 (11.5)
	PM_{10}	22.2 (11.0)	21.8 (10.7)
	$PM_{2.5}$	11.8 (7.3)	11.9 (7.5)
	SO_2	3.9 (3.5)	3.8 (3.2)

6	CO	528.6 (245.1)	545.7 (235.5)
	NO_x	29.4 (27.0)	29.0 (27.5)
	O_3	30.0 (11.8)	29.7 (11.6)
	PM_{10}	22.3 (11.1)	22.5 (11.0)
	$PM_{2.5}$	11.8 (7.3)	12.3 (7.7)
	SO_2	3.9 (3.5)	3.9 (3.6)
7	CO	529.8 (246)	545.2 (228.1)
	NO_x	29.7 (27.2)	29.2 (27.3)
	O_3	29.9 (11.8)	29.5 (11.7)
	PM_{10}	22.4 (11.1)	22.6 (11.1)
	$PM_{2.5}$	11.9 (7.4)	12.1 (7.9)
	SO_2	3.9 (3.5)	3.7 (3.2)
Trimester 1	CO	562.8 (180.3)	568.4 (158.9)
	NO_x	31.4 (19.9)	30.1 (20.2)
	O_3	29.0 (8.6)	28.7 (8.5)
	PM_{10}	22.2 (5.2)	22.0 (5.1)
	$PM_{2.5}$	11.9 (3.5)	11.9 (3.4)
	SO_2	4.1 (2.8)	3.9 (2.4)
Whole pregnancy	CO	552.7 (140.4)	559.7 (134.7)
	NO_x	30.9 (16.4)	29.5 (17.9)
	O_3	29.3 (6.2)	29.0 (6.4)
	PM_{10}	22.1 (4.3)	22.1 (4.3)
	$PM_{2.5}$	11.8 (2.8)	11.9 (3.0)
	SO_2	4.0 (2.4)	3.8 (2.2)

Abbreviations: CO, carbon monoxide; NO_x, nitric oxides; O_3, ozone; PM_{10}, particulate matter with diameter <10 microns; $PM_{2.5}$, particulate matter with diameter <2.5 microns; SO_2, sulfur dioxide.

[a]All values are expressed in µg/m^3 for particulate matter, and parts per billion for gaseous pollutants.

Supplemental Table 2. Distribution of air pollutants by exposure windows.

Exposure windows	Pollutant	Distribution[a]							
		Min	P25	P50	P75	Max	Mean	SD	IQR
Delivery day 0	CO	82.4	354.2	481.5	651.7	2694.2	529.8	246.9	297.5
	NO_x	1.8	11.3	19.6	39.0	258.3	29.7	27.4	27.7
	O_3	0.1	21.0	29.7	38.9	79.8	29.9	11.8	17.9
	PM_{10}	0.0	14.4	21.0	28.4	98.7	22.5	11.1	14.0
	$PM_{2.5}$	0.0	6.4	10.0	15.5	63.9	11.8	7.4	9.1
	SO_2	0.0	1.8	2.8	4.8	40.5	3.9	3.5	2.9
Days prior to delivery									
1	CO	82.4	353.0	481.2	650.8	2694.2	528.1	245.3	297.8
	NO_x	1.8	11.0	19.4	38.8	258.3	29.4	27.2	27.8
	O_3	0.1	21.1	29.8	38.9	79.8	30.0	11.8	17.8
	PM_{10}	0.0	14.4	21.0	28.4	98.7	22.4	11.1	14.0
	$PM_{2.5}$	0.0	6.4	10.0	15.4	63.9	11.8	7.4	9.0
	SO_2	0.0	1.8	2.8	4.7	40.5	3.9	3.5	2.9
2	CO	82.4	350.7	479.3	647.5	2694.2	526.1	245.3	296.7
	NO_x	1.8	10.8	19.0	38.0	258.3	29.0	27.0	27.2
	O_3	0.1	21.3	29.9	39.0	79.8	30.1	11.8	17.7
	PM_{10}	0.0	14.4	20.8	28.3	98.7	22.3	11.1	13.9
	$PM_{2.5}$	0.0	6.4	9.9	15.4	63.9	11.8	7.4	9.0
	SO_2	0.0	1.8	2.8	4.7	40.5	3.9	3.5	2.9
3	CO	82.4	350.4	478.0	646.6	2694.2	525.0	243.7	296.2
	NO_x	1.8	10.7	18.9	37.6	258.3	28.7	26.5	26.9
	O_3	0.1	21.3	29.9	39.0	79.8	30.1	11.8	17.7
	PM_{10}	0.0	14.3	20.7	28.1	98.7	22.2	11.0	13.9
	$PM_{2.5}$	0.0	6.4	9.9	15.3	63.9	11.8	7.3	8.9
	SO_2	0.0	1.8	2.8	4.7	40.5	3.9	3.5	2.9
4	CO	82.4	351.1	478.9	647.2	2694.2	525.4	243.5	296.1
	NO_x	1.8	10.7	18.9	37.7	258.3	28.7	26.4	27.0
	O_3	0.1	21.3	29.9	39.0	79.8	30.1	11.8	17.7
	PM_{10}	0.0	14.3	20.7	28.1	98.7	22.2	11.0	13.8
	$PM_{2.5}$	0.0	6.4	9.9	15.3	63.9	11.8	7.3	8.9
	SO_2	0.0	1.8	2.8	4.7	40.5	3.9	3.4	2.9
5	CO	82.4	352.3	479.4	647.7	2694.2	526.4	243.6	295.4
	NO_x	1.8	10.8	19.0	38.0	258.3	28.9	26.6	27.1
	O_3	0.1	21.3	29.9	39.0	79.8	30.1	11.8	17.7
	PM_{10}	0.0	14.3	20.8	28.1	98.7	22.2	11.0	13.9
	$PM_{2.5}$	0.0	6.4	9.9	15.4	63.9	11.8	7.3	8.9

	SO_2	0.0	1.8	2.8	4.7	40.5	3.9	3.5	2.9
6	CO	82.4	353.8	481.4	650.6	2694.2	528.7	245.1	296.9
	NO_x	1.8	11.1	19.4	38.8	258.3	29.4	27.0	27.7
	O_3	0.1	21.2	29.8	38.9	79.8	30.0	11.8	17.8
	PM_{10}	0.0	14.4	20.9	28.3	98.7	22.3	11.1	13.9
	$PM_{2.5}$	0.0	6.5	10.0	15.5	63.9	11.8	7.3	9.0
	SO_2	0.0	1.8	2.8	4.7	40.5	3.9	3.5	2.9
7	CO	82.4	354.2	482.1	652.9	2694.2	529.9	245.9	298.7
	NO_x	1.8	11.3	19.6	39.0	258.3	29.7	27.2	27.7
	O_3	0.1	21.1	29.7	38.9	79.8	29.9	11.8	17.8
	PM_{10}	0.0	14.4	21.0	28.4	98.7	22.4	11.1	14.0
	$PM_{2.5}$	0.0	6.5	10.0	15.5	63.9	11.9	7.4	9.0
	SO_2	0.0	1.8	2.8	4.8	40.5	3.9	3.5	2.9
Trimester 1	CO	179.1	420.2	544.8	683.8	1102.4	562.8	180.2	263.6
	NO_x	5.5	14.9	25.1	45.1	89.6	31.4	19.9	30.2
	O_3	8.1	22.8	29.2	35.1	48.7	29.0	8.6	12.3
	PM_{10}	10.3	18.6	21.6	24.9	39.2	22.2	5.2	6.3
	$PM_{2.5}$	4.6	9.3	11.9	14.5	22.0	11.9	3.5	5.3
	SO_2	0.8	2.0	3.3	5.3	18.7	4.1	2.8	3.3
Whole pregnancy	CO	224.9	449.0	547.9	655.3	1045.6	552.7	140.4	206.3
	NO_x	6.4	17.0	28.9	41.2	81.6	30.9	16.4	24.2
	O_3	13.4	24.9	28.5	32.7	46.4	29.3	6.2	7.8
	PM_{10}	10.7	19.5	22.0	23.9	39.8	22.1	4.3	4.4
	$PM_{2.5}$	5.7	9.4	11.9	14.1	19.6	11.8	2.8	4.7
	SO_2	1.0	2.1	3.3	5.0	15.8	4.0	2.4	2.9

Abbreviations: CO, carbon monoxide; NO_x, nitric oxides; O_3, ozone; PM_{10}, particulate matter with diameter <10 microns; $PM_{2.5}$, particulate matter with diameter <2.5 microns; SO_2, sulfur dioxide.

[a]All values are expressed in µg/m^3 for particulate matter, and parts per billion for gaseous pollutants.

Supplemental Table 3. Spearman correlation coefficient among pollutants during the whole pregnancy period.

Exposure window	Pollutant	CO	NO_x	O_3	PM_{10}	$PM_{2.5}$	SO_2
Delivery day 0	CO	1.00					
	NO_x	0.74	1.00				
	O_3	-0.31	-0.41	1.00			
	PM_{10}	0.21	0.27	0.08	1.00		
	$PM_{2.5}$	0.35	0.39	-0.07	0.67	1.00	
	SO_2	0.28	0.40	-0.25	0.06	0.34	1.00
Days prior to delivery							
1	CO	1.00					
	NO_x	0.74	1.00				
	O_3	-0.31	-0.41	1.00			
	PM_{10}	0.21	0.27	0.07	1.00		
	$PM_{2.5}$	0.35	0.39	-0.07	0.67	1.00	
	SO_2	0.28	0.40	-0.25	0.06	0.33	1.00
2	CO	1.00					
	NO_x	0.74	1.00				
	O_3	-0.32	-0.41	1.00			
	PM_{10}	0.21	0.27	0.07	1.00		
	$PM_{2.5}$	0.35	0.39	-0.06	0.68	1.00	
	SO_2	0.28	0.40	-0.25	0.07	0.33	1.00
3	CO	1.00					
	NO_x	0.73	1.00				
	O_3	-0.31	-0.41	1.00			
	PM_{10}	0.21	0.26	0.08	1.00		
	$PM_{2.5}$	0.35	0.38	-0.06	0.68	1.00	
	SO_2	0.28	0.40	-0.25	0.06	0.33	1.00
4	CO	1.00					
	NO_x	0.73	1.00				
	O_3	-0.31	-0.41	1.00			
	PM_{10}	0.21	0.26	0.08	1.00		
	$PM_{2.5}$	0.35	0.38	-0.06	0.68	1.00	
	SO_2	0.28	0.40	-0.25	0.06	0.33	1.00
5	CO	1.00					
	NO_x	0.73	1.00				
	O_3	-0.31	-0.41	1.00			
	PM_{10}	0.21	0.26	0.08	1.00		

	$PM_{2.5}$	0.34	0.38	-0.06	0.68	1.00	
	SO_2	0.27	0.40	-0.25	0.07	0.33	1.00
6	CO	1.00					
	NO_x	0.74	1.00				
	O_3	-0.31	-0.41	1.00			
	PM_{10}	0.21	0.26	0.09	1.00		
	$PM_{2.5}$	0.35	0.39	-0.06	0.68	1.00	
	SO_2	0.28	0.40	-0.25	0.07	0.33	1.00
7	CO	1.00					
	NO_x	0.73	1.00				
	O_3	-0.30	-0.41	1.00			
	PM_{10}	0.21	0.26	0.08	1.00		
	$PM_{2.5}$	0.35	0.39	-0.06	0.68	1.00	
	SO_2	0.28	0.40	-0.25	0.06	0.33	1.00
Trimester 1	CO	1.00					
	NO_x	0.68	1.00				
	O_3	-0.42	-0.48	1.00			
	PM_{10}	0.02	0.10	0.06	1.00		
	$PM_{2.5}$	0.28	0.40	-0.38	0.47	1.00	
	SO_2	0.27	0.38	-0.39	-0.25	0.39	1.00
Whole pregnancy	CO	1.00					
	NO_x	0.61	1.00				
	O_3	-0.26	-0.33	1.00			
	PM_{10}	0.10	0.25	-0.20	1.00		
	$PM_{2.5}$	0.25	0.47	-0.68	0.40	1.00	
	SO_2	0.31	0.38	-0.45	-0.34	0.53	1.00

Abbreviations: CO, carbon monoxide; NO_x, nitric oxides; O_3, ozone; PM_{10}, particulate matter with diameter <10 microns; $PM_{2.5}$, particulate matter with diameter <2.5 microns; SO_2, sulfur dioxide.

Supplemental Table 4. Associations between whole pregnancy exposures to criteria air pollutants and stillbirth risk by gestational week[a].

	Stillbirth	aRR (95% CI)[b]					
Gestational week	n	Ozone	CO	NO_x	PM_{10}	$PM_{2.5}$	SO_2
23-26	292	1.91 (0.94, 3.87)	1.14 (0.81, 1.62)	1.01 (0.37, 2.74)	1.07 (0.73, 1.57)	1.12 (0.57, 2.20)	0.56 (0.28, 1.15)
27-30	163	0.92 (0.41, 2.10)	1.04 (0.68, 1.61)	1.74 (0.48, 6.37)	1.29 (0.81, 2.05)	1.53 (0.66, 3.52)	1.09 (0.43, 2.73)
31-36	301	1.16 (0.74, 1.83)	0.81 (0.62, 1.07)	1.10 (0.55, 2.19)	0.71 (0.54, 0.93)	0.71 (0.40, 1.28)	0.91 (0.47, 1.77)
37-42	236	1.42 (0.95, 2.13)	0.88 (0.65, 1.19)	0.74 (0.38, 1.42)	0.82 (0.61, 1.11)	0.84 (0.44, 1.59)	0.97 (0.51, 1.85)

Abbreviations: CO, carbon monoxide; NO_x, nitric oxides; O_3, ozone; PM_{10}, particulate matter with diameter <10 microns; $PM_{2.5}$, particulate matter with diameter <2.5 microns; SO_2, sulfur dioxide; RR, relative risk; CI, confidence interval.

[a] Exposures for ongoing pregnancies were truncated to ensure comparable exposure length as stillbirth cases (e.g., average exposure up to 23 weeks as “whole pregnancy”).

[b]Models adjusted for maternal age, maternal race, parity, smoking during pregnancy, alcohol, insurance, marital status, hypertension, diabetes, season, birth year, site, and temperature. Estimates are for an IQR increase in each pollutant.

Supplemental Table 5. Multipollutant associations between whole pregnancy exposures to criteria air pollutants and stillbirth risk.

	aRR (95% CI)[a]					
Exposure windows	Ozone	CO	NO_x	PM_{10}	$PM_{2.5}$	SO_2
Days before delivery						
0 (delivery day)	1.14 (1.00 ,1.30)	0.99 (0.91 ,1.08)	0.94 (0.82 ,1.08)	0.93 (0.81 ,1.06)	1.13 (0.99 ,1.28)	1.00 (0.87 ,1.16)
1	1.14 (1.00 ,1.30)	0.97 (0.90 ,1.06)	0.92 (0.80 ,1.06)	0.93 (0.81 ,1.06)	**1.15 (1.01 ,1.31)**	1.09 (0.95 ,1.26)
2	1.13 (0.98 ,1.30)	1.01 (0.93 ,1.09)	0.98 (0.86 ,1.12)	0.93 (0.81 ,1.06)	1.00 (0.88 ,1.14)	1.09 (0.95 ,1.25)
3	1.08 (0.94 ,1.25)	1.01 (0.93 ,1.10)	1.04 (0.92 ,1.18)	0.90 (0.78 ,1.03)	0.98 (0.85 ,1.12)	1.08 (0.94 ,1.24)
4	1.13 (0.99 ,1.30)	0.99 (0.91 ,1.06)	1.02 (0.90 ,1.15)	1.00 (0.87 ,1.13)	1.04 (0.92 ,1.19)	0.99 (0.86 ,1.13)
5	**1.25 (1.09 ,1.43)**	1.01 (0.93 ,1.10)	1.04 (0.92 ,1.18)	0.99 (0.87 ,1.13)	1.04 (0.91 ,1.19)	0.89 (0.78 ,1.03)
6	**1.19 (1.04 ,1.36)**	1.04 (0.95 ,1.13)	1.07 (0.95 ,1.22)	1.00 (0.88 ,1.14)	1.02 (0.90 ,1.17)	0.95 (0.83 ,1.08)
7	**1.15 (1.00 ,1.31)**	0.95 (0.87 ,1.03)	1.04 (0.91 ,1.19)	0.97 (0.85 ,1.12)	1.06 (0.92 ,1.21)	1.01 (0.88 ,1.16)
First trimester	1.12 (0.92 ,1.37)	**0.76 (0.62 ,0.92)**	1.34 (0.95 ,1.88)	1.11 (0.93 ,1.33)	0.86 (0.69 ,1.06)	**0.74 (0.61 ,0.91)**
Whole pregnancy	1.32 (1.00 ,1.76)	0.89 (0.70 ,1.13)	1.50 (0.84 ,2.68)	1.03 (0.79 ,1.33)	1.02 (0.62 ,1.68)	0.75 (0.47 ,1.21)

Abbreviations: RR, relative risk; CI, confidence interval; CO, carbon monoxide; NO_x, nitric oxides; PM_{10}, particulate matter with diameter <10 microns; $PM_{2.5}$, particulate matter with diameter <2.5 microns; SO_2, sulfur dioxide.

Bold face indicates statistical significance at $p<0.05$.

[a]Models adjusted for maternal age, maternal race, parity, smoking during pregnancy, alcohol, insurance, marital status, hypertension, diabetes, season, birth year, site, and temperature. Estimates are for an IQR increase in each pollutant.

Supplemental Table 6. Adjusted[a] relative risk (and 95% confidence intervals) for stillbirth per IQR unit increase in pollutant by exposure window and asthma status.

Pollutant and asthma status		Days before delivery							
		0	1	2	3	4	5	6	7
CO	Asthma	0.85 (0.63,1.16)	0.97 (0.74,1.26)	0.81 (0.64,1.03)	0.92 (0.73,1.16)	0.88 (0.70,1.12)	0.92 (0.67,1.25)	0.96 (0.76,1.22)	0.90 (0.67,1.21)
	No asthma	0.91 (0.77,1.07)	0.98 (0.85,1.13)	0.87 (0.77,1.00)	0.91 (0.80,1.04)	0.94 (0.82,1.08)	0.94 (0.80,1.11)	0.98 (0.86,1.12)	0.95 (0.81,1.11)
NO_X	Asthma	0.98 (0.76,1.27)	1.13 (0.89,1.43)	0.88 (0.70,1.09)	0.91 (0.72,1.15)	0.87 (0.67,1.14)	0.98 (0.75,1.28)	0.98 (0.79,1.22)	0.93 (0.74,1.18)
	No asthma	1.00 (0.87,1.16)	1.09 (0.95,1.24)	0.92 (0.81,1.04)	0.93 (0.81,1.06)	0.94 (0.81,1.10)	0.97 (0.83,1.13)	0.99 (0.88,1.13)	0.97 (0.85,1.11)
O_3	Asthma	0.99 (0.70,1.40)	0.94 (0.69,1.28)	1.01 (0.73,1.39)	1.02 (0.75,1.38)	0.96 (0.70,1.30)	1.09 (0.77,1.55)	1.20 (0.84,1.70)	1.30 (0.94,1.81)
	No asthma	1.04 (0.86,1.27)	1.02 (0.85,1.22)	1.09 (0.91,1.31)	1.08 (0.91,1.29)	1.04 (0.87,1.24)	1.16 (0.95,1.40)	1.19 (0.97,1.44)	1.21 (1.00,1.46)
SO_2	Asthma	0.97 (0.82,1.16)	1.04 (0.86,1.25)	1.06 (0.89,1.26)	1.03 (0.86,1.22)	1.00 (0.84,1.19)	1.10 (0.93,1.30)	1.14 (0.95,1.37)	0.96 (0.78,1.18)
	No asthma	0.98 (0.89,1.09)	1.02 (0.92,1.13)	1.03 (0.94,1.14)	1.02 (0.93,1.13)	1.00 (0.90,1.10)	1.04 (0.95,1.15)	1.09 (0.98,1.21)	0.96 (0.86,1.08)
PM_{10}	Asthma	0.85 (0.67,1.08)	0.96 (0.75,1.24)	1.01 (0.77,1.31)	1.05 (0.81,1.35)	1.00 (0.78,1.29)	1.03 (0.78,1.37)	1.14 (0.87,1.49)	0.98 (0.71,1.35)
	No asthma	0.92 (0.81,1.05)	1.01 (0.88,1.16)	1.04 (0.90,1.20)	1.07 (0.93,1.22)	1.01 (0.88,1.16)	0.99 (0.85,1.16)	1.08 (0.94,1.25)	1.01 (0.85,1.20)
$PM_{2.5}$	Asthma	0.96 (0.77,1.21)	0.96 (0.75,1.23)	1.02 (0.81,1.29)	1.12 (0.91,1.39)	1.10 (0.90,1.34)	1.10 (0.87,1.40)	1.17 (0.93,1.48)	1.02 (0.77,1.34)
	No asthma	0.97 (0.85,1.10)	0.99 (0.87,1.13)	1.03 (0.91,1.17)	1.09 (0.97,1.22)	1.06 (0.95,1.18)	1.05 (0.92,1.19)	1.12 (0.98,1.27)	1.04 (0.90,1.20)

Abbreviations: RR, relative risk; CI, confidence interval; CO, carbon monoxide; NO_x, nitric oxides; PM_{10}, particulate matter with diameter <10 microns; $PM_{2.5}$, particulate matter with diameter <2.5 microns; SO_2, sulfur dioxide.

[a]Models adjusted for maternal age, maternal race, parity, smoking during pregnancy, alcohol, insurance, marital status, hypertension, diabetes, season, birth year, site, and temperature. Estimates are for an IQR increase in each pollutant.
